# Multiple regimes of robust patterns between network structure and biodiversity

**DOI:** 10.1038/srep17856

**Published:** 2015-12-03

**Authors:** Luis F. Jover, Cesar O. Flores, Michael H. Cortez, Joshua S. Weitz

**Affiliations:** 1 School of Physics, Georgia Institute of Technology, Atlanta, GA, USA; 2 Department of Mathematics and Statistics, Utah State University, Logan, UT, USA; 3 School of Biology, Georgia Institute of Technology, Atlanta, GA, USA

## Abstract

Ecological networks such as plant-pollinator and host-parasite networks have structured interactions that define who interacts with whom. The structure of interactions also shapes ecological and evolutionary dynamics. Yet, there is significant ongoing debate as to whether certain structures, e.g., nestedness, contribute positively, negatively or not at all to biodiversity. We contend that examining variation in life history traits is key to disentangling the potential relationship between network structure and biodiversity. Here, we do so by analyzing a dynamic model of virus-bacteria interactions across a spectrum of network structures. Consistent with prior studies, we find plausible parameter domains exhibiting strong, positive relationships between nestedness and biodiversity. Yet, the same model can exhibit negative relationships between nestedness and biodiversity when examined in a distinct, plausible region of parameter space. We discuss steps towards identifying when network structure could, on its own, drive the resilience, sustainability, and even conservation of ecological communities.

Ecological communities are often composed of a large number of interacting types, e.g., species, morphs or strains. Interaction patterns in a community can be represented as a network where nodes denote distinct types and links between nodes denote connections between individuals of the respective types. These connections can represent distinct modes of ecological interactions including predation, mutualism, competition and parasitism. Understanding the relationship between network structure and the subsequent dynamics of populations has, for decades, been facilitated by theory. For example, Robert May’s seminal work in the early 1970s introduced the idea that large complex networks were more likely to be unstable^1^,^2^. Whether or not complexity begets instability in an ecological community remains controversial, as May’s original conclusions depend, in part, on assumptions regarding the choice of the underlying interactions and *random* network structure^3^,^4^,^5^,^6^,^7^.

In reality, ecological interactions are both complex and structured. Realized networks may differ in terms of their connectance, nestedness and modularity - as but three examples of differentiating features. Connectance is defined as the ratio of realized links to potential links; nestedness quantifies the extent to which there exists a hierarchy such that interaction ranges of increasingly specialized types are organized as proper subsets of the interaction ranges of more generalist types^8^; and modularity quantifies the extent to which organisms tend to interact within densely connected groups rather than between groups^9^,^10^. Network features, including connectance, modularity, and nestedness^11^,^12^,^13^ have been shown, in theory, to affect the biodiversity and stability of the underlying community^11^,^14^.

For example, Bascompte and colleagues showed that mutualistic plant-pollinator networks tend to be nested and conjectured that nestedness increases biodiversity, quantified in terms of the relative fraction of surviving types in a dynamic model^14^. Yet, in contrast, James and colleagues^15^ argued that nestedness is a covariate of, rather than a driving factor for, increases in biodiversity. Similarly, Suweis *et al.* showed that elevated nestedness can emerge as a consequence of adaptations that increase species-level and total community abundance^16^. As a second example, phage-bacteria infection networks are often significantly nested^13^. Phage-bacteria communities with nested networks have been shown to be stable^17^, so long as there are trade-offs between interaction ranges and other life history traits. Nestedness may even facilitate the emergence of increased biodiversity of both bacteria and phage given a single limiting resource for bacterial growth when contrasted to the dynamics of bacteria-only environments^18^.

How is theory used to study the relationship between network architecture and biodiversity? By and large, theoretical studies typically represent an ecological community via a system of differential equations that describe the change in the population abundances of interacting types. In some instances, it is possible to determine the relationship between network architecture and biodiversity^18^. Yet, when analytical solutions are not available, then many studies select model parameters from biologically plausible, prior distributions and simulate system dynamics to identify statistical relationships between network structure and biodiversity (see Fig. 1 and 11,15,19). Such approaches raise a question: how does the selection of the prior parameter distribution influence model dynamics and the resulting relationship between network structure and biodiversity? Further, is it possible that there are distinct, robust relationships between network structure and biodiversity given different parameter assumptions?

In this manuscript, we examine the entanglement of network architecture and model parametrization and their combined effect on biodiversity (Fig. 1). To do so, we simulate ensembles of models given distinct assumptions of plausible life history traits. We find that the relationship between patterns of network architecture and ecological dynamics can vary qualitatively with model parameterization. Throughout, we focus on a specific model of virus-bacteria dynamics and study the relationship between one ecological property – community biodiversity, i.e., the fraction of surviving strains – and one network property – nestedness. Nonetheless, we explain how our findings can be applied to other systems in which there is uncertainty with respect to the life history traits of interacting strains.

## Results

### A rule-based framework to identify distinct domains of biodiversity-nestedness relationships

We are interested in studying coexistence of multiple strains in virus-bacteria systems and its relationship to model parametrization. We define biodiversity as the fraction of surviving strains in the system and simulate the interactions between strains using the model described in the Methods. In general, coexistence of all the strains in a system, i.e., a biodiversity value of 1, requires the existence of a steady state with positive densities for all strains. For a given interaction matrix, we refer to a steady state with positive densities for all strains as a feasible steady state and to the associated parameter set as a feasible parameter set.

The mathematical conditions for feasibility can be formulated in terms of the parameters of a model: *r*, bacterial growth rate; *K*, carrying capacity; *a*, bacterial competition; as well as *ϕ*, *m*, and *β*; denoting virus adsorption rate, decay rate, and burst size respectively. As an example, consider a low-dimensional system with two bacteria, two viruses, and a nested infection network (virus 1 infects bacteria 1 and 2 and virus 2 infects only bacteria 1). To simplify the analysis we assume that virus adsorption rates and burst sizes are independent of bacterial strains (i.e. *ϕ*_*ij*_ = *ϕ*_*j*_, *β*_*ij*_ = *β*_*j*_) and that intra-specific and inter-specific competition between bacterial strains is equal (i.e. *a*_*ii*′_ = 1). Under these assumptions the conditions for feasibility are: *r*_1_ > *r*_2_ and 
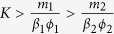
. These conditions represent a feasible “volume” in the 9-dimensional parameter space, i.e., a region of parameter space where coexistence of all 4 strains is possible. Further, we see that the condition for feasibility involving growth rates divides the corresponding two-dimensional parameter subspace into two areas: a feasible region where coexistence of all strains is possible(*r*_1_ > *r*_2_), and an infeasible region where coexistence of all strains is not possible (*r*_2_ < *r*_1_). As should be evident, randomly sampling parameter space would affect conclusions regarding the total potential biodiversity in the system.

**Figure 2:**
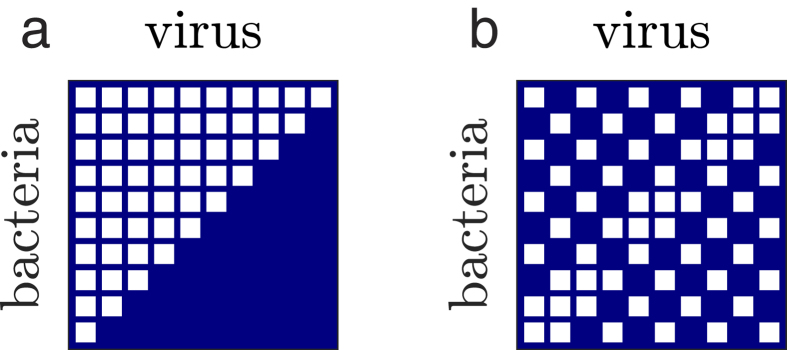
Focal matrices used in the rule-based framework. (**a**) Low nested network (NODF = 0.21). (**b**) Perfectly nested network (NODF = 1). White cells denote that a virus in that column is able to infect the bacteria in that row, whereas blue cells denote the absence of infection.

We can extend the analysis of this low-dimensional example to the case of a perfectly nested infection network with 10 bacteria and 10 viruses (Fig. 2a). The rules for feasibility are an extension of the rules from the previous 2 virus, 2 bacteria example, namely: 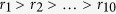 and 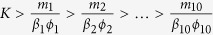. These rules can be generalized for a perfectly nested network of arbitrary size with equal number of bacterial and virus strains^17^. In the case of 10 bacteria and 10 viruses, the rules define a feasible region in the 41-dimensional parameter space of the model. We sample parameters from two different regions of parameter space to illustrate the effect of model parameterization on biodiversity-nestedness relationships. First, we sample parameters from the feasible region of the perfectly-nested network (Fig. 2a) where all strains coexist when the interaction matrix is nested. Second, we sample parameters from the feasible region of a low-nestedness network (Fig. 2b); details of the network and its feasible region are presented in the Methods for the rule-based framework.

**Figure 3:**
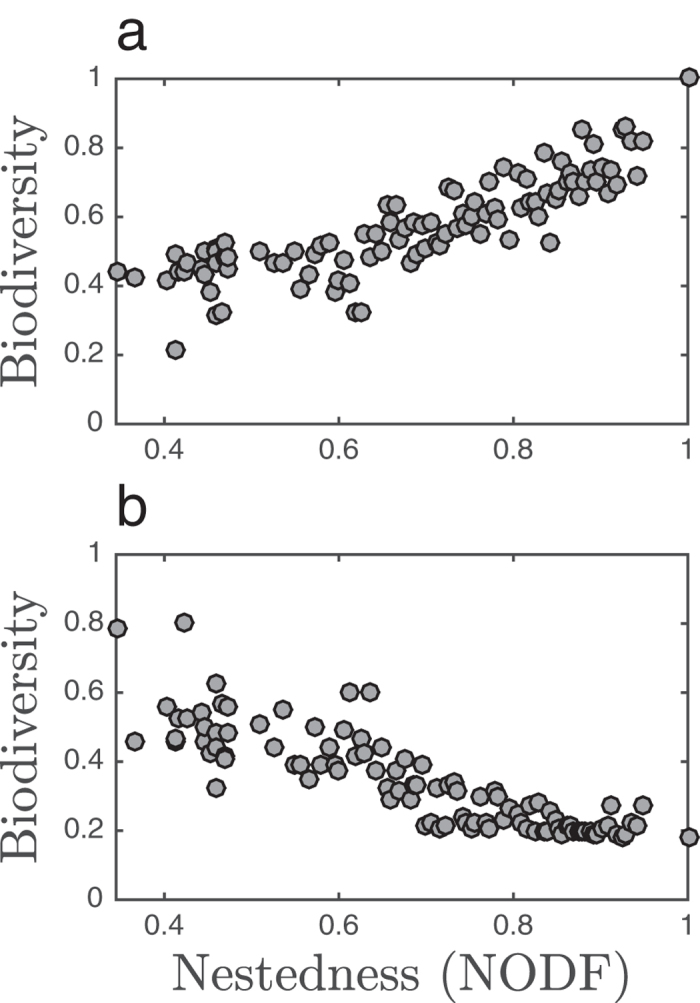
Average biodiversity over 100 different sets of parameters for 100 different matrices spanning nestedness values from 0.35 to 1. (**a**) Parameter sets sampled from the feasible region of the perfectly nested networks (Table 1). (**b**) Parameter sets sampled from the feasible region of a low-nestedness network (Table 2).

Given the sampling regions, numerical simulations of model dynamics are averaged over an ensemble of 100 infection networks representing a gradient in nestedness while conserving the connectance of each network in the ensemble. When parameters are chosen from the feasible region of the perfectly nested network (Table 1), we observe a positive biodiversity-nestedness relationship (Fig. 3a). In contrast, when parameters are chosen from the feasible region of a low-nestedness network (Table 2), we see a negative biodiversity-nestedness relationship (Fig. 3b). The perfectly nested network was included in the ensemble of 100 networks. Sampling parameters from its feasible region resulted in survival of all the strains in the system (average biodiversity of 1, Fig. 3a). The low-nestedness network used to obtain the second sampling region was not included in the ensemble because it has a different connectance than the perfectly nested network and the rest of the networks. We found that the trends in biodiversity were robust to changes in initial conditions when sampling values from biologically plausible regions. The key point of this analysis is that the relationship between biodiversity and nestedness differs qualitatively when examining two distinct feasible sets of parameters.

### A feasibility-based framework to identify distinct domains of biodiversity-nestedness relationships

**Figure 4:**
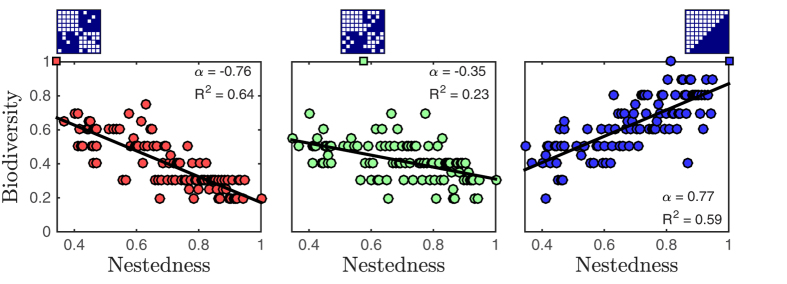
For fixed parameter values in different regions of parameter space, the relationship between biodiversity and nestedness can be positive, negative, or exhibit a weak trend. Each panel corresponds to a different (fixed) parameterization of the model. Each point represents the biodiversity for a particular interaction matrix whose nestedness (NODF) lies between 0.35 and 1. The matrix for which biodiversity is maximized is plotted above each panel. (Left, red) A negative biodiversity-nestedness relationship arises when the parameter set maximizes biodiversity for a network with low nestedness (NODF = 0.35). (Middle, green) A weaker negative trend arises when the parameter set maximizes biodiversity for network with intermediate nestedness (NODF = 0.57). (Right, blue) A positive biodiversity-nestedness relationship arises when the parameter set maximizes biodiversity for a network with high nestedness (NODF = 1). The slope of a linear fit *α* and coefficient of determination *R*^2^ are presented (*p* < 10^−5^ for all the fitted lines).

For a general interaction network the conditions for feasibility are more complicated than the conditions presented for the perfectly-nested network derived in the previous section. As a consequence, we developed an alternative framework for selecting parameter sets that maximize biodiversity in Eqs. (1)-(2), 19 for any non-trivial infection network. This alternative framework does not require finding the rules for feasibility explicitly. Instead, we choose a subset of the parameters randomly from a biologically plausible region (Table 3), specify the target steady state densities of the bacteria and viruses, and then solve for the rest of the parameters using the steady state equations. In this way it is possible to obtain a particular feasible parameter set for any infection network for which all nodes have at least one link (see Methods for more details). We selected three particular parameter sets for which biodiversity is maximized for three different infection networks of 10 bacteria and 10 viruses. We calculated average biodiversity for an ensemble of 100 matrices which included the three focal networks (Fig. 4). Figure 4 shows that not only is it possible to maximize biodiversity for different networks, but it is also possible that the resulting trends are qualitatively different. We show that, by maximizing biodiversity for a low-nestedness matrix, we obtain a negative trend of biodiversity vs. nestedness (Fig. 4 left). In contrast we obtain a positive trend of biodiversity vs. nestedness by selecting parameters that maximize biodiversity for a perfectly nested networks (Fig. 4 right). It is also possible that the quantitative strength of the relationship can differ, approaching the case where the is no significant relationship between biodiversity and nestedness (Fig. 4 middle). This analysis supports the prior conclusions, i.e., that biodiversity-nestedness relationships depend on model parameterization.

### Biodiversity-nestedness relationships are robust to local perturbation of parameter sets

**Figure 5:**
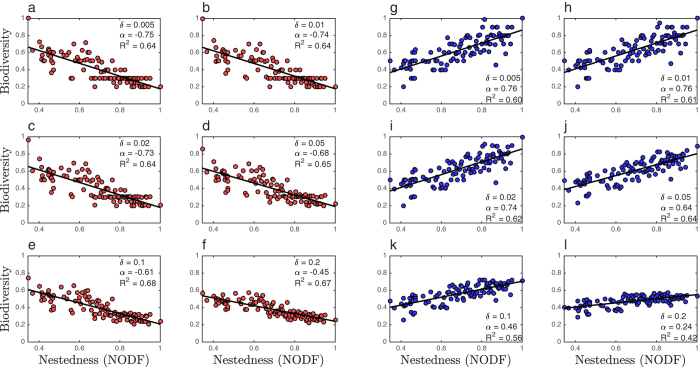
Biodiversity-nestedness relationships are robust to local perturbations of parameter values. (Panels (**a**–**f**) red) Average biodiversity for 100 parameter sets sampled from a uniform distribution centered around a parameter set that maximizes biodiversity for a low-nestedness network. (Panels (**g**–**l**) blue) Average biodiversity for 100 parameter sets sampled from a uniform distribution centered around a parameter set that maximizes biodiversity for a perfectly nested network. Each plot corresponds to a different value of *δ* which determines the size of the region used for sampling. The slope of a linear fit *α* and coefficient of determination *R*^2^ are presented (*p* < 10^−5^ for all the fitted lines).

Here, we examine biodiversity-nestedness relationships when parameters and network structure are both varied. To do so, we sampled parameter values from regions of parameter space centered about the original parameter set which maximized biodiversity for a particular interaction network (as explained in the previous section). The size of the region was set by the parameter *δ* such that for a given value of a parameter, 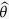, the random parameter values were sampled from the interval 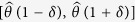. Here *θ* is a dummy parameter denoting any of the life history traits, *r*, *K*, *m*, *β* and *ϕ* that impact Eqs. (1)-(2), 19. Figures 5(a–f) show evidence for a robust, negative biodiversity-nestedness relationship that persists even as the interval width increases. We see that the negative trend is robust to selecting parameters from a region, but the trend becomes weaker as the size of the intervals are increased. Similarly, Figures 5(g–l) show evidence for a robust, positive biodiversity-nestedness relationship observed in a different area of parameter space that also persists as the sampling interval is increased. In summary, qualitatively distinct biodiversity-nestedness relationships persist, i.e., are robust, when parameter values are chosen at random from a region of parameter space.

## Discussion

We analyzed a nonlinear model of phage-bacteria dynamics (Eqs. (1)-(2), 19) as a means to investigate the entanglement between interaction network structure, life history traits, and biodiversity. Given the complexity in the space of possible networks, we considered ensembles of networks that varied in a particular structural feature – nestedness – such that interaction ranges differ in the extent to which they form partially ordered subsets of one another. We found that there is not one globally applicable, positive relationship between biodiversity and nestedness in this model (Fig. 3). Instead, we identified distinct regions in parameter space where there are contrasting relationships, both positive and negative (Figs 4 and 5).

Elevated nestedness is a common feature of interaction networks spanning both plant-pollinator and phage-bacteria systems^12^,^13^. Moreover, prior theoretical work has suggested that ecological communities whose interaction networks are nested are more likely to have higher relative biodiversity^12^. Our results highlight the need to understand the life history context underlying a given biodiversity-nestedness relationship. The totality of parameter space includes a subspace of biologically plausible values. Such subspaces often have relatively uninformative prior distributions. Therefore, using biologically plausible regions to restrict the parameters of a model is not a strong enough restriction to uniquely define the effect of network structure on community dynamics.

**Figure 6:**
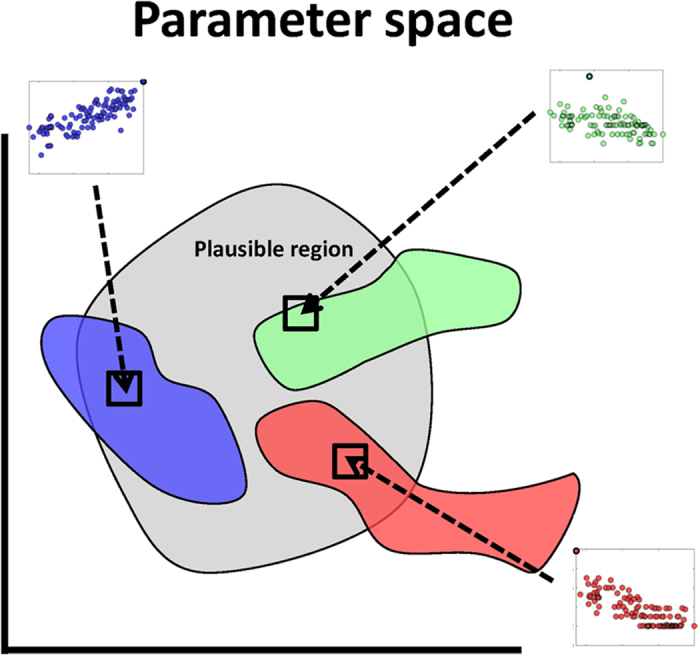
Schematic representation of parameter space: biologically plausible region and feasible regions for different infection networks. Black squares represent sampling regions that result in different trends of biodiversity vs. nestedness.

Recently, it has been pointed out that different model parameterizations can lead to different biodiversity levels and consequently to contradictory results^20^. We expand on this point to show that this is also the case for whole regions of parameter space. This is important because studies using numerical simulations often average over different parameterizations. Indeed, Rohr *et al.*^20^ examine to what extent parameters can vary for a given network structure before the community make-up changes. In contrast, our work examines how changes in network structure affects community make-up for a fixed set (or region) of parameters. These approaches are related, but they are not the same. Our results show how averaging over parameterizations is not sufficient to account for the effects of life history traits and that a more systematic study of parameter space is necessary. Additionally, we make a stronger case for the effect of parametrization by showing that it is possible to not only maximize biodiversity for specific networks but to obtain completely different trends of biodiversity vs. nestedness (Fig. 6).

In our view, statistical inference from numerical simulations can be informative and even advantageous, so long as certain precautions are kept in mind. The key point is that systematic analysis of parameter space is necessary whenever a numerical approach is used to characterize network structure-biodiversity relationships in nonlinear ecological systems. Studies that rely on analytical methods to estimate biodiversity or related features usually focus on fixed-point equilibrium states that are stable either locally or globally. This could be problematic for two reasons. First, general analytical solutions could be hard to find or interpret. Second, coexistence in high-dimensional ecological models could be characterized by non-equilibrium steady states. In such circumstances, fixed-point analyses would overlook configurations that are ecologically relevant.

In the case of phage-bacteria dynamics, our study highlights the value of additional measurements of life history traits, complementary to the recent focus on methods for characterizing who infects whom^21^. We used a particular phage-bacteria model to highlight the importance of systematically studying model parametrization in distinct regions to better understand the relationship between network structure and biodiversity - yet the findings are relevant to a wider debate. The current findings point to the need to revisit the relationship between network structure, life history traits and biodiversity in other systems and given other kinds of network patterns. Optimistically, the systematic study of model parametrization could be of service in resolving ongoing debates concerning the relationship, or relationships, between biodiversity and network structure in plant-pollinator systems^14^,^15^,^20^,^22^.

## Methods

### Model

**Figure 7:**
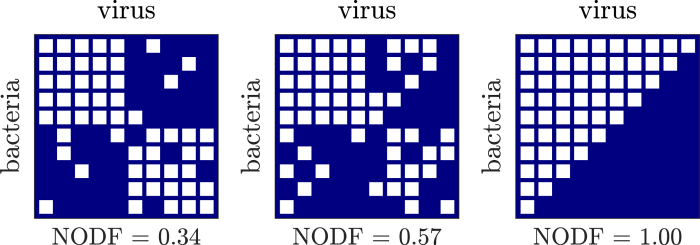
Focal matrices used in the feasibility-based framework. The left-most network has low nestedness and the rightmost network has high nestedness. Each row represents the interactions of a given bacteria type with all viruses and each column represents the interaction of a given virus type with all bacteria. White cells denote instances in which a given virus in column *j* infects a given bacteria in row *i* (i.e., *M*_*ij*_ = 1), whereas blue cells denote the lack of infection (i.e., *M*_*ij*_ = 0).

The dynamics of virus-bacteria systems can be modeled using systems of coupled, nonlinear differential equations^23^,^24^. Here we use a system of equations that extend the basic Lotka-Volterra equations^25^,^26^ to incorporate multiple types of virus and bacteria and competition between bacteria^17^:


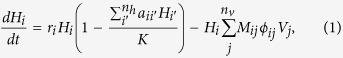



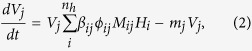


where there are *n*_*h*_ bacteria types, each with density *H*_*i*_ and *n*_*v*_ virus types, each with density *V*_*j*_. In this system: *r*_*i*_ is the growth rate of bacteria *i* in the absence of phage and other bacteria, *a*_*ii*′_ is the competitive effect of bacteria *i*′ on bacteria *i* (set to 1 for the analysis), *K* is the system-wide carrying capacity, *ϕ*_*ij*_ is the adsorption rate of phage *j* when attaching to bacteria *i*, *β*_*ij*_ is the effective burst size of phage *j* when infecting bacteria *i*, and *m*_*j*_ is the decay rate of virus *j*. Finally the element *M*_*ij*_ denotes which virus infects which bacteria such that *M*_*ij*_ = 1 if bacteria *i* is infected by virus *j* and is zero otherwise; altogether these interactions can be represented as a network (Fig. 7) where each type is equivalent to a strain^27^. We are interested in identifying fixed points where all strains have positive densities, such that:


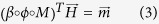



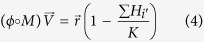


We refer to these points as feasible fixed points. In the present model the feasible fixed point is the solution 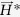 and 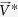 to Eqs. (3) and (4). Here, 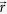 and 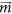 are vectors whose elements are the growth and decay rates and *β*, *ϕ*, and *M* are matrices whose elements are the *β*_*ij*_, *ϕ*_*ij*_, and *M*_*ij*_ respectively; “O” denotes element-wise matrix multiplication.

### Network ensemble

We generated ensembles of networks with fixed connectance and fixed size, *n*_*h*_ × *n*_*v*_ = *n*^2^, given the further assumption that *n* = *n*_*v*_ = *h*_*h*_. The ensemble generation procedure builds upon that introduced by James and colleages^15^. Starting with a perfectly nested network, randomly chosen interactions are removed from the existing interactions among bacteria and viruses and an equal number of new interactions are added among bacteria-virus pairs where interactions did not exist originally. In the conventional matrix representation this is akin to random removal of interactions in the top left corner of the matrix followed by an equivalent addition of new interactions in the bottom right corner of the matrix. Figure 7 shows an example of three matrices with different values of nestedness generated using this procedure. To generate a large ensemble of matrices with varying degrees of nestedness the number of randomly selected links that where moved was varied from 1 to (*n*/2)^2^. We selected 100 invertible matrices for our ensemble and used the Non-overlapping and Decreasing Fill (NODF) metric for nestedness^8^.

### Estimating biodiversity from numerical simulation of the dynamics

Biodiversity is defined in this study as the fraction of surviving strains in Eqs. (1)-(2), 19 after a sufficiently long transient. We estimated biodiversity by numerically integrating the system using MATLAB’s ODE45 and enumerating the number of strains with densities greater than 10^−10^ ml at the end of the simulation. We used this criteria for survival instead of relying on stability analysis of equilibria (or other attractors) because strain coexistence could occur via a stable fixed point, periodic cycles, or chaotic dynamics.

The stopping time of the simulation was determined via a heuristic that evaluates the convergence of time-averaged densities and mitigates inconsistencies introduced by arbitrarily choosing a stopping time. In developing the heuristic, we leveraged the fact that the average density of each strains is equal to its equilibrium density in Lotka-Volterra systems. The algorithm to determine stopping time is as follows:

- Every 500 hours calculate the infection matrix of only those strains with densities greater than 10^−10^.
- If the subsystem is solvable (invertible community matrix) calculate the theoretical interior fixed point.
- Check if the average density over the last half of the simulation is within 10 percent of the theoretical prediction of the subsystem.
- The simulation stops when the last condition is satisfied or the simulation has run for 40000 hours.

**Figure 8:**
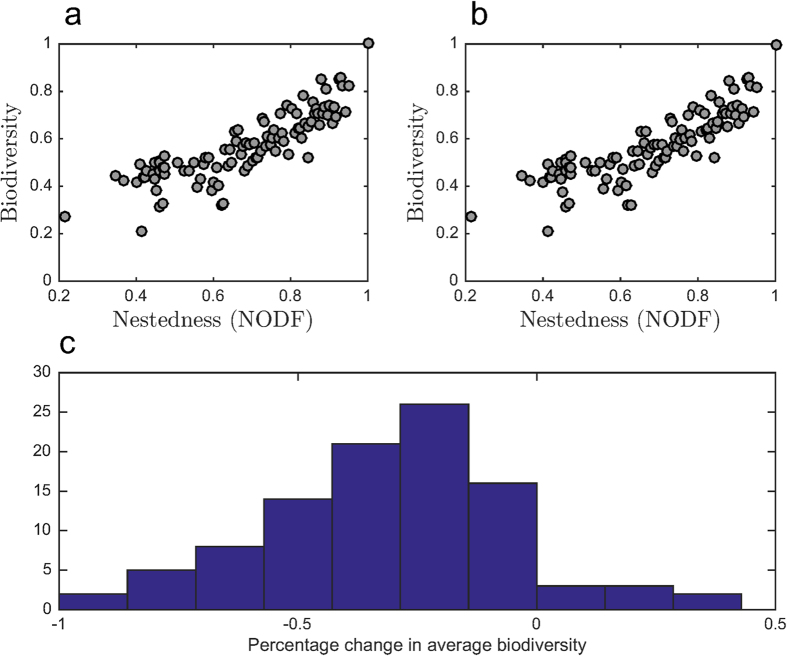
Doubling the time of the simulations obtained with the stopping time heuristic results in less than a 1% change in the computed average biodiversity. (**a**) Average biodiversity using the stopping time heuristic. (**b**) Average biodiversity using double the time used in (**a**). Percentage change between (**a**,**b**).

Note that the algorithm only calculates the stopping time and does not alter the densities of the strains. To evaluate the robustness of the stopping criteria, we calculated persistence using the stopping time heuristics and compared the results with ones obtained after doubling the time for all simulations. Figure 8 shows the relationship between persistence and nestedness and the difference in mean persistence. Note that the relationship between biodiversity and nestedness are the same whether using the heuristic stopping time or twice this value (Fig. 8a,b). We find that there are less than 1% differences in the average biodiversity between the two stopping times (Fig. 8c). Altogether, the heuristic captures the long term coexistence of different strains even when the dynamics do not tend to a steady state.

### Parameter range selection

We use two different frameworks to select parameter ranges. In doing so, we use the term “plausible” to denote those parameter sets and steady-state densities whose values are consistent with virus-host biology and ecology. In addition, we use the term “feasible” to denote those parameter sets whose associated steady state densities are all positive for a given infection matrix. The steps to generate parameter sets in each of the frameworks are:

Ruled-based framework:

- Choose a focal matrix.
- Solve for the feasible steady state of the focal matrix implicitly in terms of model parameters.
- Choose plausible regions in parameter space that satisfy the feasible steady state conditions.
- Generate a parameter set by sampling uniformly from feasible and biologically plausible regions.

Feasibility-based framework:

- Choose a focal matrix.
- Sample one set of values each of carrying capacity, adsorption rate, burst size, virus and host steady state densities (*K*, *ϕ*_*j*_, *β*_*j*_, 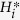, 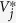) for all species from biologically plausible regions.
- Solve the steady state equations to find specific values of host growth rate and virus mortality rate, *r*_*i*_ and *m*_*i*_, respectively for all species.
- If the values of *r*_*i*_ and *m*_*j*_ are plausible, then these together with the previously sampled *K*, *ϕ*_*j*_, and *β*_*j*_ form a feasible and plausible parameter set.
- Generate a parameter set by sampling uniformly from a region centered around the specific feasible parameter set.

We used three focal networks in the feasibility-based framework (Fig. 7) and two focal networks in the ruled-based framework (Fig. 2). The following sections explain each framework in detail.

#### Parameter range selection in the ruled-based framework

In the rule-based framework, we start with a particular focal matrix and analytically solve for the steady states. From the steady states we obtain constraints on the life history traits (rules in the form of inequalities) which guarantee feasibility. For example, feasibility in the perfectly nested network requires an ordering of host growth rates 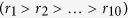 amongst other rules. We then choose biologically plausible regions of parameter space that satisfy these rules and look at how biodiversity varies with network nestedness. For this, we draw uniformly distributed parameters from these fixed regions and calculate average persistence for an ensemble of networks that span a large range of nestedness values. We followed this framework for two different focal matrices: one with high nestedness (Fig. 2b) and one with low nestedness (Fig. 2a). This framework has the advantage that the rules for feasibility characterize the entirety of parameter space by dividing it into feasible and non-feasible regions for each focal network. Nonetheless, it is not trivial to generalize this framework to an arbitrary focal network. As a consequence we were not able to find a low-nestedness network with the same connectance as the rest of the matrix in the ensemble and with simple feasibility rules. Instead, the focal low-nestedness network used to show a different trend of persistence vs. nestedness has a slightly larger connectance (three more interactions) than the rest of the matrices in the ensemble.

#### 1. Feasibility conditions for the perfectly nested network

We make the following assumptions regarding Eqs. (1)–(2): there is equal intra-specific and inter-specific competition across bacterial strains (*a*_*ii*′_ = 1), and virus adsorption rates and burst sizes are independent of bacterial strains (*ϕ*_*ij*_ = *ϕ*_*j*_, *β*_*ij*_ = *β*_*j*_). Using these assumptions we find general expressions for the steady states, H^*^ and V^*^, in terms of the life history traits for a 10 by 10 perfectly nested network (Fig. 2b) by solving Eqs. (3) and (4):


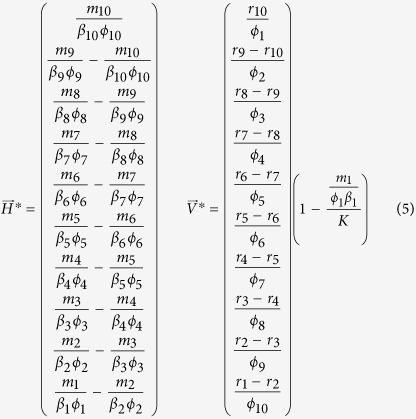


In order for all strains to have positive densities, the life history traits must satisfy:


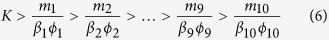



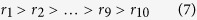


These expressions generalize to perfectly nested networks of any size^17^.

#### 2. Feasibility conditions for the low-nestedness network

Here we derive constraints on the life history traits for a low-nestedness network. In order to obtain rules that are easier to interpret we set *a*_*ii*′_ = 1, *β*_*ij*_ = *β*, and *ϕ*_*ij*_ = *ϕ*. These assumptions imply that: intra-specific and inter-specific competition between bacterial strains is equal and the virus burst size and adsorption rates are independent of host and virus strain. We use the low-nestedness matrix presented in Fig. 2a, which has several symmetries that result in simpler rules. By solving Eqs. (3) and (4) with this matrix we find the following steady states:


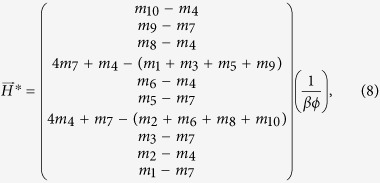



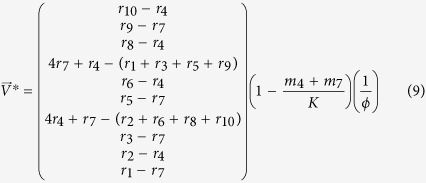


In order for all strains to have positive densities, the life history traits must satisfy:


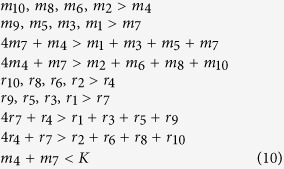


#### Parameter range selection in the feasibility-based framework

In this framework parameters are selected from intervals centered around particular feasible parameter sets of a focal network. We make the following assumptions to the general model (Eqs. (1)–(2)): equal intra-specific and inter-specific competition across bacterial strains (*a*_*ii*′_ = 1), and virus adsorption rates and burst sizes are independent of bacterial strains (*ϕ*_*ij*_ = *ϕ*_*j*_, *β*_*ij*_ = *β*_*j*_). These assumptions are not necessary to implement this framework, but we use them because they simplify the analysis. In particular, under these assumptions, a feasible coexistence equilibrium is guaranteed to exist whenever the infection matrix *M* is invertible. When these assumptions are not satisfied, one must check whether the matrices *β* o *ϕ* o *M* and *ϕ* o *M* are invertible; defined in Eqs. (3) and (4). If the system is not solvable then the life history traits generated contain degeneracies that make some combination of viral or host strains effectively equal. We selected three focal infection networks *M* (Fig. 7), and values *K*, *ϕ*_*j*_, *β*_*j*_, H^*^, V^*^ from random uniform distributions in biologically plausible regions. Then, using Eqs. (3) and (4), we obtained values for *m*_*i*_ and *r*_*i*_. With this procedure we obtain a set of life history traits values that yield a feasible fixed point. For each parameter value, *θ*, local perturbations are made by sampling from an interval of length 2*δθ* centered around *θ*. The particular values of delta used are included in the caption of Fig. 5.

## Additional Information

**How to cite this article**: Jover, L. F. *et al.* Multiple regimes of robust patterns between network structure and biodiversity. *Sci. Rep.* **5**, 17856; doi: 10.1038/srep17856 (2015).

## Figures and Tables

**Figure 1:**
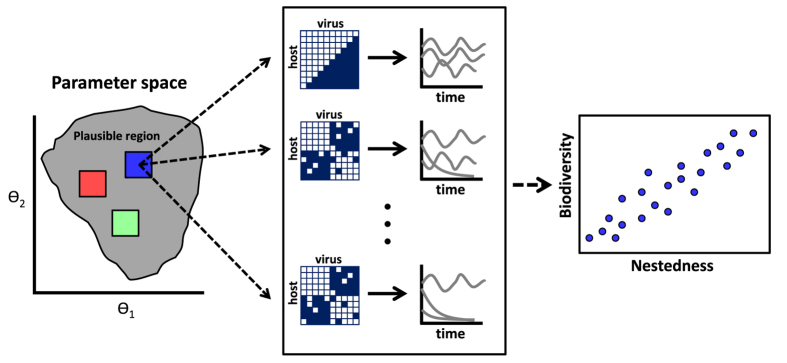
Schematic of how studies using numerical simulations have drawn insights into the relationship between network structure and biodiversity. (Left panel) A focal region of parameter space is examined (the blue square, in the parameter space of *θ*_1_ − *θ*_2_). (Middle panel) Given parameter variation in a focal region, the network is modified across a spectrum of configurations, here from low to high nestedness from bottom to top (where white cells denote interactions and blue cells denote the absence of interactions). Then, the dynamics of each system are simulated and/or analyzed given variation in network structure and model parameters. The proportion of surviving species in the simulations determines the biodiversity of each community. (Right panel) Variation in resulting biodiversity is compared to the variation in network structure, e.g., nestedness (right panel). Given the large number of parameters, such studies do not exclude the possibility that distinct structure-biodiversity relationships may exist for different life history parameters (the red and green boxes in the left panel).

**Table 1:** Parameter ranges used to obtain feasible parameter sets for the perfectly nested network.

Parameter (units)	Range\Value
*r*_*i*_ (1/d)	3.61–43.35
*ϕ*_*j*_ (ml/(virus · d))	2.4 × 10^−7^–2.4 × 10^−6^
*β*_*j*_ (virus/cell)	10–100
*m*_*j*_ (1/d)	0.037–0.52
*K* (ml)	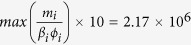

Parameters were sampled from uniform distributions and sorted according to the rules of feasibility for the perfectly nested network presented in rule-based framework of the Results. The limits of the distributions are specified in the column titled Range. A fixed value was used for *K*.

**Table 2:** Parameter ranges used to obtain feasible parameter sets for the low-nestedness network.

Parameter (units)	Range\Value
*r*_*i*_ for *i* = 1, 2, 3, 5, 6, 8, 9, 10 (1/d)	4.06–4.51
*r*_*i*_ for *i* = 4, 7 (1/d)	3.61–4.06
*ϕ*_*j*_ (ml/(virus · d))	1.32 × 10^−6^
*β*_*j*_ (virus/cell)	30
*m*_*j*_ for *j* = 1, 2, 3, 5, 6, 8, 9, 10 (1/d)	0.47–0.52
*m*_*j*_ for *j* = 4, 7 (1/d)	0.42–0.47
*K* (ml)	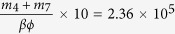

Range denotes the limits of the uniform distribution used to generate parameters. The limits were calculated such that the parameters satisfy the rules of feasibility for the low-nestedness network presented in the feasibility-based framework as shown in the Methods. Fixed values were used for *ϕ*, *β*, and *K*.

**Table 3:** Parameter and target steady state density ranges used in the feasibility-based framework.

Parameter (unit)	Range\Value
*ϕ*_*j*_ (ml/(viru · d))	2.4 × 10^−8^–2.4 × 10^−7^
*β*_*j*_ (viruses/cell)	10–100
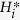 (cell/ml)	10^3^–10^4^
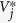 (virus/ml)	10^6^–10^7^
*K* (ml)	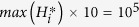

Bacteria growth rates, *r*_*i*_, and virus decay rates, *m*_*j*_, were derived using the steady state equations and the parameters presented in this table (see Methods, given feasibility-based framework). The range denotes the limits of the uniform distributions used to generate parameters.

## References

[b1] May R. M. Will a large complex system be stable? Nature 238, 413–414 (1972). 4559589 10.1038/238413a0

[b2] May R. M. Stability and complexity in model ecosystems vol. 6 (Princeton University Press, 2001).

[b3] Roberts A. The stability of a feasible random ecosystem. Nature 251, 607–608 (1974).

[b4] Allesina S. & Pascual M. Network structure, predator–prey modules, and stability in large food webs. Theor. Ecology 1, 55–64 (2008).

[b5] Bascompte J. *et al.* Disentangling the web of life. Science 325, 416 (2009). 19628856 10.1126/science.1170749

[b6] Allesina S. & Tang S. Stability criteria for complex ecosystems. Nature 483, 205–208 (2012). 22343894 10.1038/nature10832

[b7] Loreau M. From populations to ecosystems: Theoretical foundations for a new ecological synthesis (MPB-46), 10.1515/9781400834167 (Princeton University Press, 2010).

[b8] Almeida-Neto M., Guimaraes P., Guimarães P. R., Loyola R. D. & Ulrich W. A consistent metric for nestedness analysis in ecological systems: reconciling concept and measurement. Oikos 117, 1227–1239 (2008).

[b9] Newman M. E. Modularity and community structure in networks. P. Natl. Acad. Sci. USA 103, 8577–8582 (2006). 10.1073/pnas.0601602103 PMC1482622 16723398

[b10] Barber M. J. Modularity and community detection in bipartite networks. Phys. Rev. E 76, 066102 (2007). 10.1103/PhysRevE.76.066102 18233893

[b11] Thébault E. & Fontaine C. Stability of ecological communities and the architecture of mutualistic and trophic networks. Science 329, 853–856 (2010). 20705861 10.1126/science.1188321

[b12] Bascompte J., Jordano P., Melián C. J. & Olesen J. M. The nested assembly of plant–animal mutualistic networks. P. Natl. Acad. Sci. USA 100, 9383–9387 (2003). 10.1073/pnas.1633576100 PMC170927 12881488

[b13] Flores C. O., Meyer J. R., Valverde S., Farr L. & Weitz J. S. Statistical structure of host–phage interactions. P. Natl. Acad. Sci. 108, E288–E297 (2011). 10.1073/pnas.1101595108 PMC3136311 21709225

[b14] Bastolla U. *et al.* The architecture of mutualistic networks minimizes competition and increases biodiversity. Nature 458, 1018–1020 (2009). 19396144 10.1038/nature07950

[b15] James A., Pitchford J. W. & Plank M. J. Disentangling nestedness from models of ecological complexity. Nature 487, 227–230 (2012). 22722863 10.1038/nature11214

[b16] Suweis S., Simini F., Banavar J. R. & Maritan A. Emergence of structural and dynamical properties of ecological mutualistic networks. Nature 500, 449–452 (2013). 23969462 10.1038/nature12438

[b17] Jover L. F., Cortez M. H. & Weitz J. S. Mechanisms of multi-strain coexistence in host–phage systems with nested infection networks. J. Theor. Biol. 332, 65–77 (2013). 23608631 10.1016/j.jtbi.2013.04.011

[b18] Korytowski D. A. & Smith H. L. How nested and monogamous infection networks in host-phage communities come to be. Theor. Ecol. 8, 111–120 (2014).

[b19] Saavedra S., Stouffer D. B., Uzzi B. & Bascompte J. Strong contributors to network persistence are the most vulnerable to extinction. Nature 478, 233–235 (2011). 21918515 10.1038/nature10433

[b20] Rohr R. P., Saavedra S. & Bascompte J. On the structural stability of mutualistic systems. Science 345, 1253497 (2014). 25061214 10.1126/science.1253497

[b21] Tadmor A. D., Ottesen E. A., Leadbetter J. R. & Phillips R. Probing individual environmental bacteria for viruses by using microfluidic digital PCR. Science 333, 58–62 (2011). 21719670 10.1126/science.1200758 PMC3261838

[b22] Saavedra S. & Stouffer D. B. “Disentangling nestedness” disentangled. Nature 500, E1–E2 (2013). 23969464 10.1038/nature12380

[b23] Levin B. R., Stewart F. M. & Chao L. Resource-limited growth, competition, and predation: a model and experimental studies with bacteria and bacteriophage. Am. Nat. 3–24 (1977).

[b24] Abedon S. T. Bacteriophage ecology: population growth, evolution and impact of bacterial viruses, 10.1017/CBO9780511541483 (Cambridge University Press, Cambridge, UK).

[b25] Lotka A. J. Elements of Physical Biology, 92–94 (Williams & Wilkins Company, Baltimore, USA, 1925).

[b26] Volterra V. Fluctuations in the abundance of a species considered mathematically. Nature 118, 558–560 (1926).

[b27] Weitz J. S. *et al.* Phage–bacteria infection networks. Trends Microbiol. 21, 82–91 (2013). 23245704 10.1016/j.tim.2012.11.003

